# Nurses at the Front Line of COVID-19: Roles, Responsibilities, Risks, and Rights

**DOI:** 10.4269/ajtmh.20-0650

**Published:** 2020-08-11

**Authors:** Mirna Fawaz, Huda Anshasi, Ali Samaha

**Affiliations:** 1 Nursing Department, Faculty of Health Sciences, Beirut Arab University, Beirut, Lebanon;; 2 School of Nursing, The University of Jordan, Amman, Jordan;; 3 Department of Biomedical Sciences, Lebanese International University, Beirut, Lebanon

Nurses have critical roles and responsibilities during the COVID-19 pandemic. They will continue to be at the front line of patient care in hospitals and actively involved with evaluation and monitoring in the community. Nurses have to ensure that all patients acquire personalized, high-quality services irrespective of their infectious condition. They will also engage in planning for anticipated COVID-19–related outbreaks, which increase the demand for nursing and healthcare services that might overload systems.^1^ Moreover, nurses must maintain effective supply and usage of sanitation materials and personal protective equipment and offer screening information, confinement guidelines, and triage protocols based on the latest guidance. A global pandemic needs strong nursing staff engagement in clinical management, awareness and knowledge exchange, and public safety.

The American Nurses Association’s Code of Ethics for Nurses (2015) is the definitive professional conduct norm for the nursing field. Clause 2 of the code specifies that “the sole responsibility of the nurse is toward the patient.” Clause 5 of the code notes that the nurse has the same obligation to themselves and to others. During outbreaks, these equitable responsibilities can clash as nurses have to constantly care for contagious patients, especially in pressing situations with scarce or unavailable resources and unrestrained contagion. Nurses and their coworkers will have to determine how much care they could give to others in times of pandemics, while still taking care of themselves.^2^

As nurses are at the front line of the COVID-19 outbreak response and are exposed to hazards that put them at risk of infection, it is vital that they are supported to protect themselves with specific infection prevention procedures and sufficient provision of protective gear at their practice settings, including ventilators, masks, robes, eye cover, face shields, and gloves.^3^ Nursing managers and instructors must include guidance to nurses and support personnel on emerging COVID-19 problems and hazards that are unique to their field of work.

Currently, there has been substantial confusion about the methods of transmission of COVID-19, who is at risk of spreading or catching the virus, and where spreads originate.^4^ These misconceptions may circulate across mainstream media, on social networking platforms or in society, and they can conflict with attempts to respond to public health issues. Nurses hold a vital function, as one of the most distinguished health service teams, in delivering public awareness regarding disease prevention and in decreasing the dissemination of myths regarding the epidemic. This involves countering myths, guiding people to available health services, and supporting evidence-based patient management and infection reduction initiatives.^5,6^

Nurses are now actively involved in COVID-19 interventions, and they will remain key players in stopping the pandemic with adequate assistance. Thus, they must be provided with a healthy work environment to empower their efforts to control and manage the outbreak. Such a work environment should be a judgment-free atmosphere for staff, where they will feel free to comment on accidents such as exposure to body fluids, other infection control risks, or reports of abuse, and to take prompt follow-up action such as providing counseling for staff members. First and foremost, occupational safety is key to nurses’ work during COVID-19, as they are face-to-face with danger on a daily basis. The overarching duty of nurse leadership will be to ensure that the appropriate prevention and security steps are taken to reduce the dangers of the workplace. In this respect, it is important that hospitals have appropriate infection control procedures and personal protective equipment (masks, gloves, goggles, gowns, hand antiseptics, soap and water, and cleaning materials) in ample amounts for personnel who care for suspect or verified COVID-19 patients. Nursing supervisors should offer knowledge on workplace security, in addition to instruction and guidance regarding infection prevention and control and how to properly don, doff, and discard personal protective equipment. In this regard, staff members should also be guided on how to carry out regular self-assessments, and directed on how to follow quarantine or isolation measures, when indicated, to protect them, their families, and their community, as well as to safeguard their mental health and well-being.^7^

As for the contractual side of this issue, nursing managers and supervisors are expected to enable nurses to assert the right to withdraw from a job arrangement if they have fair reasons to conclude that their assignments require a significant threat to their life or safety. If a health worker practices this privilege, then they should be shielded from any adverse effects. Furthermore, nurses should be owed the right to reimbursement, psychological counseling, and therapeutic care if they are diagnosed with COVID-19 through contact at work.
